# Pathways to Prosociality: How Classroom Strategies That Support Basic Psychological Needs Foster Prosocial Behavior in Children

**DOI:** 10.1007/s11121-026-01895-2

**Published:** 2026-03-18

**Authors:** Yue Sun, Amanda W. G. van Loon, Tessa M. L. Kaufman

**Affiliations:** 1 University of Amsterdam, Research Institute for Child Development and Education, Nieuwe Achtergracht 127, 1018 WS Amsterdam, the Netherlands; 2 Child and Adolescent Studies, Utrecht University, Heidelberglaan 1, 3584 CS Utrecht, the Netherlands

**Keywords:** Prosocial behavior, Classroom-based intervention, Basic Psychological Needs Theory, Meaningful roles, Compliments, Democratic classroom meetings

## Abstract

This study investigated how specific components of a classroom-based intervention promote prosocial behavior in primary school children, guided by *Basic Psychological Needs Theory* (BPNT). Drawing on data from a large-scale intervention (*N* = 1132, 43.3% girls, *M*_age_ = 9.96, *SD*_age_ = 1.22), we examined two sequential mediation pathways: (1) the *quantity* pathway: whether the presence of intervention components (meaningful roles, peer compliments, and democratic classroom meetings) predicted prosocial behavior through increased general need fulfilment (autonomy, competence, relatedness) and prosocial motivation; (2) the *quality* pathway: whether the specific basic psychological needs fulfilment within each component predicted prosocial behavior through increased general need fulfilment and prosocial motivation. Results supported both mediation pathways. For the quantity pathway, the presence of compliments and democratic classroom meetings showed indirect effects through general autonomy and relatedness, whereas meaningful roles exerted a primarily direct influence. For the quality pathway, component-specific relatedness emerged as the most consistent predictor of prosocial behavior, followed by more selective effects for component-specific autonomy, namely, only for meaningful roles. This suggests that everyday classroom strategies and experiences can incrementally contribute to general need fulfillment, thereby promoting prosocial behavior. These findings underscore the importance of identifying specific mechanisms of interventions and highlight how need-supportive strategies can foster prosocial development in school settings.

**Supplementary Information:**

The online version contains supplementary material available at 10.1007/s11121-026-01895-2.

Teaching children to behave prosocially—to voluntarily act in ways that promote others' well-being (Eisenberg et al., 2015)—is a key aspect of socioemotional education (Jackson et al., 2020). Prosocial behavior, such as sharing, helping, and cooperating, has been linked to increased happiness and reduced stress in the short term (Aknin et al., 2012; Poulin & Holman, 2013), while long-term benefits include improved physical health and greater life satisfaction (Caprara & Steca, 2005; Okun et al., 2013). Further, prosociality stems from a broader commitment to social justice and equips children for civic participation (Caprara et al., 2015), ultimately contributing to a more cooperative society. Thus, promoting prosocial behavior in children benefits both individuals and society, short- and long-term.

To foster prosocial behavior in children, intervention strategies often target classroom settings, where children spend a substantial amount of time with peers and encounter opportunities for prosocial actions (Freitas et al., 2021). It is widely acknowledged that enhancing prosociality is most effective when *intrinsic* motivation is fostered, given the inherently voluntary nature of such actions (Eisenberg et al., 2015). Intrinsic motivation refers to doing something because it is interesting or enjoyable, rather than doing something because of external pressure or rewards (i.e., extrinsic motivation; Ryan & Deci, 2000a). While existing evidence supports the efficacy of classroom-based prosocial interventions (e.g., Freitas et al., 2021; Mesurado et al., 2019), it remains unclear *why* they are effective—specifically, how individual intervention components contribute to these processes. Addressing this gap, the present study examined how individual components of a classroom-based intervention support *Basic Psychological Needs* (BPNs), considered fundamental to (intrinsic) prosocial motivation, and how they subsequently promote prosocial behaviors – through prosocial motivation for showing such behaviors – in primary school students.

## Fulfilment of Basic Psychological Needs to Enhance Prosocial Behavior

*Basic Psychological Need Theory* (BPNT), one of the six mini-theories within Self-Determination Theory (Ryan & Deci, 2000b), offers a robust framework for promoting intrinsic motivation to act prosocially. It posits that intrinsic motivation depends on the fulfilment of three fundamental needs: autonomy (the sense of volition and willingness), competence (the feeling of effectiveness and confidence in one’s abilities), and relatedness (feeling connected; Ryan & Deci, 2000b). Satisfying these needs promotes growth-oriented tendencies, which, given humans’ inherently social nature, often manifest as positive social activity, including prosocial motivation and behavior (Cheon et al., 2019). Notably, the fulfilment of these needs does not require major accomplishments or life-changing events; even minor, everyday interactions, such as helping a friend, can enhance competence and relatedness (Ryan, 2023). This reveals the potential of leveraging need fulfilment through simple, intentional actions.

## Intervention Components

Interventions informed by basic psychological needs have generally been found effective in fostering positive changes in need fulfillment and enhancing physical and mental well-being (Cheon et al., 2019; Ntoumanis et al., 2021). However, limited research has explored the *mechanisms* by which individual intervention components or strategies of these programs produce such effects. This study focuses on three key intervention components in the Dutch Meaningful Role (SterkWerk) program: (1) providing students *meaningful roles* to emphasize responsibility and collaboration, (2) encouraging students to exchange *compliments* to build connections and confidence, and (3) facilitating *democratic classroom meetings* to promote mutual understanding and involvement. Collectively, these components seek to create a supportive environment in which students can fulfill fundamental needs and improve social relationships (van Loon & Kaufman, 2023).

### Meaningful Roles

Providing students with meaningful classroom roles gives them responsibilities that foster accountability and elevate their social status through prosocial contributions (Ellis et al., 2016). From an evolutionary perspective, antisocial behaviors such as bullying may serve as strategies for gaining social status and access to resources (Ellis et al., 2012; Volk et al., 2014). Rather than solely addressing such behaviors through reactive approaches, a potentially more effective strategy is to offer socially valued opportunities for gaining recognition and social connection (Ellis et al., 2016). Through this approach, educators can functionally encourage prosocial behaviors. Schools with a higher proportion of students in meaningful roles have been found to exhibit more positive environments and reduced conflicts (Ellis et al., 2016). Taking on responsibilities within the classroom also requires students to be seen as reliable by their peers (Wray-Lake & Syvertsen, 2011), encouraging meaningful contributions within their sphere of influence. Such responsibility has been linked to the fulfillment of basic psychological needs—autonomy, competence, and relatedness—which in turn predicts increases in prosocial behavior (Manzano-Sánchez et al., 2021).

### Compliments

Another core intervention component involves growth-oriented compliments which reinforce students’ positive behaviors through social recognition. Compliments serve as positive reinforcement, increasing the likelihood that specific behaviors will be repeated (Ellis et al., 2016). As children enter late childhood and early adolescence, they become increasingly sensitive to peer influence (Lease et al., 2020), they quickly learn which behaviors lead to peer acceptance, and compliments from classmates can therefore shape their behavioral choices. It is therefore particularly effective to encourage timely, specific compliments on performance or effort. These growth-oriented compliments have been shown to increase intrinsic motivation and belief in one’s capacity for behavioral improvement (Platte et al., 2025), thereby fostering a sense of competence. Over time, the exchange of compliments contributes to supportive school environments, which are associated with an increased sense of belonging (Vieno et al., 2007). Interventions utilizing peer compliment strategies have demonstrated improvements in prosocial behavior and reductions in disruptive behavior (Collins et al., 2020). In sum, growth-oriented compliments are expected to contribute to the fulfilment of competence and relatedness needs, subsequently contributing to more prosocial behavior.

### Democratic Classroom Meetings

In addition to the aforementioned components, which focus on daily peer interactions, democratic classroom meetings provide a structured opportunity to cultivate collective responsibility (Reeve & Jang, 2006). These meetings equip students to become independent problem solvers, allowing them to gain a sense of competence by practicing social skills and receiving peer support (Gfroerer et al., 2024). Such meetings fundamentally enhance autonomy by offering a platform for students to express opinions, share perspectives, and participate in decision-making. They may also strengthen the classroom community and foster relatedness among peers (Angell, 2004; Vaz et al., 2015). Empirical evidence suggests that democratic classroom meetings enhance peaceful, respectful environments and increase positive behaviors such as sharing responsibility (Pavidis, 2023). Thus, given their role in fostering autonomy, competence, and relatedness, democratic classroom meetings are expected to fulfil all three needs and subsequently, promote prosocial behavior.

## The Current Study

This study addressed a critical gap in understanding *how* one classroom-based intervention contributes to prosocial behavior by focusing on the mechanisms of specific intervention components, rather than evaluating program effects holistically. Using the Dutch Meaningful Roles program, we examined how the intervention’s three components (i.e., meaningful roles, compliments, and democratic classroom meetings) foster prosocial behavior in primary school students (aged 8–12) through the fulfilment of autonomy, competence, and relatedness needs (van Loon & Kaufman, 2023).

**Fig. 1:**
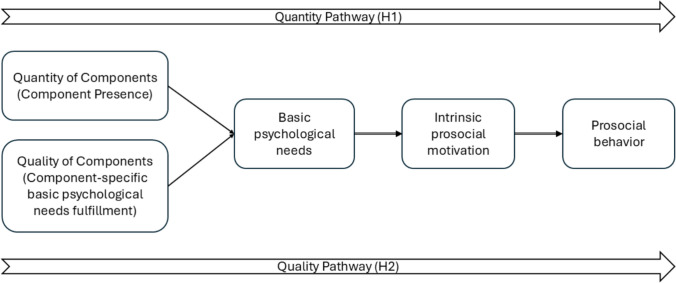
Conceptual model and hypotheses illustration

The study aimed to identify the active components and their underlying mechanisms to inform both theory and educational practice. Conceptually, we differentiated between *how often* students engaged with each intervention component (quantity) and *how good* these experiences felt in terms of need fulfilment (quality). We hypothesized two complementary pathways. First (H1: the *quantity pathway*), we expected that a higher *presence* of the three components (meaningful roles, peer compliments, and democratic classroom meetings) would predict higher levels of children’s *general* autonomy, competence, and relatedness. In turn, more general need fulfilment was expected to be associated with stronger prosocial motivation and, ultimately, more prosocial behavior. Second (H2: the *quality pathway*), we expected a greater extent to which each component fulfilled their autonomy, competence, and relatedness needs, would generalize to more overall need fulfilment, which would again relate to higher levels of prosocial motivation and subsequent prosocial behavior. See Fig. 1 for the conceptual model for the present study.

## Methods

### Procedures

This study utilized data from the “SterkWerk” program, i.e., the Dutch Meaningful Role program (van Loon & Kaufman, 2023), a cluster randomized controlled trial (RCT). Consent forms were collected from May 2023 until the start of the first assessment. Participating schools introduced the program to parents and children through brochures and informational letters, including consent forms for parents and children aged 12 years or older. Participants could submit their consent forms either on paper or electronically. The original RCT included three waves of data collection, with children and teachers completing questionnaires. For the current study, only student data from the intervention group were used, specifically from the second assessment (T2; February–March 2024) and final assessment (T3; May–June 2024). The RCT was registered at ClinicalTrials.gov (NCT05891067), and the study protocol was approved by the Ethics Committee of Utrecht University (23–0082).

### Intervention Components

The intervention consisted of three components. First, students were provided meaningful roles contributing to the class, such as homework monitors (telling absent students about missed homework) or plant technicians (watering plants). While the goals of the roles were mostly predefined, students could execute them according to their preferences. Second, growth-oriented compliments were encouraged. Students were taught to give effective compliments—specific to behavior, timely, personal, and respectful. Compliments were often made visible, for example, via a “compliments tree.” Third, democratic classroom meetings promoted student-led discussions and decision-making (e.g., how things are going in the classroom, discussing children’s input, such as compliments or suggestions). Students took on formal roles (e.g., chairperson, secretary) and led the meetings independently. For example, students could discuss having a pet in the classroom (e.g., fish) and vote for a decision.

### Participants and Missing Data

The intervention group included 20 schools, 76 classes, and 1193 students. After excluding 61 students with missing data on all study variables, the final sample consisted of 19 schools, 71 classes, and 1,132 students. Of these, 43.3% identified as girls, 45.6% as boys, and 11.1% identified otherwise or preferred not to disclose. At T2, the average age was approximately 10 years (*M* = 9.96 years, *SD* = 1.22 years). Descriptives per school for each variable are reported in the Supplementary Information 1 (Table S1). Little’s MCAR test indicated data were not likely missing *completely* at random (MCAR), χ^2^/df = 7.38 > 3, χ^2^ (277) = 2049.625 (Bollen, 1989). Thus, data were considered missing at random (MAR) and were handled using full information maximum likelihood (FIML).

### Measurements

#### Intervention Components Quantity: Component Presence

The quantity of intervention components was assessed using questionnaires specifically developed for the study at T2. The quantity was operationalized and measured by the presence, i.e., the frequencies of students’ engagement in the intervention components during the first half of the intervention (i.e., from T1-T2). We mainly used single-item measures, as most constructs regarding the intervention components are unidimensional, clearly defined, and narrow (Allen et al., 2022). Moreover, research in organizational psychology demonstrated that for many constructs, single-item measures are reliable and valid measures (Matthews et al., 2022).

**Meaningful Roles**. To assess the presence of meaningful roles, participants were asked, “Did you have a role in the past few months?”, ranging from 1 (I had no role) to 5 (I had more than 4 roles). As having more roles did not necessarily mean more effort was put in (the amount of work was not consistent across roles), this was recoded into two categories: 0 (no role) and 1 (one or more roles). Students with roles were asked, “How often were you working with the role(s)?” Responses from 1 (multiple times a day) to 6 (never) were reverse-coded. “Never” responses were merged with “no role” to yield a final range from 0 (no role or never worked with the role) to 5 (worked with the role(s) multiple times a day).

**Compliment**. The presence of compliments was measured with the item “I have received compliments from my classmates,” with responses ranging from 1 (multiple times a day) to 6 (never), and was reverse-coded to keep consistent with the directions of the other items.

**Democratic Classroom Meetings***.* The presence of the democratic classroom meetings was assessed by the item “Have you had any democratic class meetings (“Klassenvergaderingen”) in the past few months?” with answers ranging from 1 (no meetings) to 5 (one meeting per week).

#### Intervention Components Quality: Component-Specific BPN Fulfilment

The intervention components quality was also measured at T2. Students reported the extent to which each component fulfilled their basic psychological needs (BPNs) during the first half of the intervention (i.e., from T1-T2), with responses ranging from 1 (never) to 4 (always). These need-fulfillment constructs were likewise assessed with single-item indicators for the same reasons as component presence.

**Meaningful Roles-Specific BPN Fulfilment.** Measured by “I chose how I carried out my role(s)” (autonomy); “I was good at carrying out my role(s)” (competence); and “I feel that I have contributed to a pleasant class/school with my role(s)” (relatedness).

**Compliment-Specific BPN Fulfilment***.* Measured by “I felt more confident in myself when I received a compliment” (competence) and “I felt more connected/close to my classmates when I received a compliment” (relatedness).

**Democratic Classroom Meeting-Specific BPN Fulfilment.** Measured by “The meeting was done independently by children (autonomy)”; “I made my views known during the meeting” (competence); and “If someone brought something up during the meeting, that child was listened to carefully” (relatedness).

#### General BPN Fulfilment

At T2, students also rated their general BPN fulfilment at the present moment, irrespective of the intervention. Mean scores were computed for need, with higher scores indicating greater fulfilment.

**Autonomy**. Measured with five items (e.g., “In this classroom, you can choose how you want to do something”), adapted from the research de Vreedzame School (Battistich et al., 1997; Pauw, 2013) and the Perceived Choice and Awareness Scale (Sheldon et al., 1996). Items were rated from 1 (never) to 4 (always). The combined scale showed acceptable internal consistency (α = .707).

**Competence.** Measured with five items (e.g., “I think positively about myself”) from the Dutch version of the Rosenberg Self-Esteem Scale (RSES; Franck et al., 2008), rated from 1 (not true) to 3 (definitely true). RSES showed sufficient psychometric properties in adolescents (Cong & Cheong, 2023), also in the current sample (α = .781).

**Relatedness.** Measured with six items from the Classroom Peer Context Questionnaire (CPCQ; Boor-Klip et al., 2014, 2017), rated from 1 (not true) to 5 (totally true). This included four comfort items (e.g., “I belong to the group”) and two cohesion items (e.g., “Everyone likes each other”). The combined scale showed good internal consistency (α = .880). 

#### Prosocial motivation

Prosocial motivation was measured with four items specifically developed for this study (e.g., “I think it is important to help someone” or “I want to know how to be a good friend”), rated on a 3-point scale ranging from 1 (not true) to 3 (definitely true). The internal consistency was acceptable in the current sample (α = .707 for T2). Mean scores were computed, with higher scores reflecting more (intrinsic) prosocial motivation.

#### Prosocial Behavior

Prosocial behavior was assessed at T3 using four items (e.g., “I try to be nice to my classmates”) from the prosocial subscale of the Dutch SDQ (van Widenfelt et al., 2003), rated from 1 (never) to 4 (always). This version of the SDQ has demonstrated acceptable reliability and validity in youth samples (van Widenfelt et al., 2003). Mean scores were computed, with higher scores reflecting more prosocial behavior (α = .689).

### Analytical Plan

A series of path models was conducted in *Mplus* 8.1 (Muthén & Muthén, 1998-2017) to test sequential indirect associations from intervention components to prosocial behavior through basic psychological need fulfilment and prosocial motivation. General BPNs at T2 and prosocial motivation at T2 served as mediators; prosocial behavior at T3 was the outcome. Independent variables (i.e., component presence and component-specific BPN fulfilment) were also assessed at T2 but referred to experiences from the first half of the intervention (i.e., from T1-T2). Model 1 tested H1, including all three components’ presence as predictors. Models 2a–2c tested H2, with each model focusing on an individual component’s BPN fulfilment (Model 2a: meaningful roles, Model 2b: compliments, Model 2c: democratic classroom meetings). T1 prosocial behavior was included as a covariate in all models to adjust for the possibility that students with higher initial levels of prosociality may have been more willing to engage in the intervention activities and reported higher levels of need fulfilment and prosocial motivation.

To account for clustering within classes, the Mplus “Type = Complex” option was used, with class specified as the clustering variable (Maydeu-Olivares, 2017). The Maximum Likelihood Robust (MLR) estimator addressed non-normality and data non-independence. Model fit was evaluated using the Comparative Fit Index (CFI ≥ .95, acceptable ≥ .90), Root Mean Square Error of Approximation (RMSEA ≤ .05, acceptable ≤ .08), and Standardized Root Mean Square Residual (SRMR ≤ .08; Hu & Bentler, 1999). This study has been preregistered: https://osf.io/dkgsm, see Supplementary Information 2 for the deviations from the preregistration.

After estimating the final models, we tested specific indirect effects using the “MODEL INDIRECT” command in *Mplus*. In Model 1, we computed nine sequential indirect paths (p1–p9, quantity pathway), representing all combinations of the three component-presence predictors with the three general BPN mediators and the sequential path to prosocial behavior via prosocial motivation. In Models 2a–2c, we estimated eight sequential indirect paths from component-specific needs fulfilment to their corresponding general autonomy, competence, and relatedness, and subsequently to prosocial motivation and prosocial behavior (p10–p17, quality pathway).

## Results

### Descriptive Statistics

Overall, positive correlations were found between the presence of intervention components or their specific BPN fulfilment and prosocial motivation/behavior, as well as between general BPNs and prosocial motivation/behavior. Detailed descriptive statistics and correlation parameters are presented in Table 1.

**Table 1:** Descriptive statistics and correlations for study variables

Variable	Min	Max	M	SD	1	2	3	4	5	6	7	8	9	10	11	12	13	14	15	16	17
1. MR	0	5	2.58	1.86	-																
2. MR aut	1	4	2.75	1.00	.059	-															
3. MR com	1	4	3.21	0.80	.161^**^	.320^**^	-														
4. MR rel	1	4	2.84	1.00	.111^*^	.259^**^	.405^**^	-													
5. CM	0	4	2.69	1.56	.221^**^	.120^**^	.122^**^	.143^**^	-												
6. CM aut	1	4	2.80	0.94	.135^**^	.201^**^	.207^**^	.263^**^	.263^**^	-											
7. CM com	1	4	2.96	0.76	.132^**^	.250^**^	.269^**^	.286^**^	.149^**^	.251^**^	-										
8. CM rel	1	4	3.11	0.99	.108^**^	.215^**^	.252^**^	.291^**^	.116^**^	.326^**^	.352^**^	-									
9. Co	0	4	2.64	1.56	.049	.037	.045	.149^**^	-.064	.055	.164^**^	.127^**^	-								
10.Co com	1	4	3.05	0.93	.093^*^	.128^**^	.186^**^	.266^**^	.028	.126^**^	.241^**^	.201^**^	.122^**^	-							
11. Co rel	1	4	2.86	0.97	.061	.213^**^	.203^**^	.242^**^	.017	.171^**^	.300^**^	.205^**^	.148^**^	.539^**^	-						
12. General aut	1	4	2.94	0.53	.109^**^	.251^**^	.285^**^	.335^**^	.209^**^	.287^**^	.314^**^	.432^**^	.189^**^	.215^**^	.245^**^	-					
13. General com	1	3	2.59	0.42	.061	.168^**^	.229^**^	.273^**^	.084^*^	.115^**^	.231^**^	.242^**^	.209^**^	.208^**^	.177^**^	.348^**^	-				
14. General rel	1	5	4.05	0.75	.145^**^	.174^**^	.244^**^	.374^**^	.147^**^	.251^**^	.245^**^	.391^**^	.211^**^	.235^**^	.292^**^	.572^**^	.527^**^	-			
15. Pros mot	1	3	2.65	0.38	.131^**^	.309^**^	.245^**^	.109^**^	.159^**^	.208^**^	.185^**^	.101^**^	.154^**^	.157^**^	.287^**^	.324^**^	.186^**^	.330^**^	-		
16. Pros beha T1	1	4	2.80	0.50	.247^**^	.229^**^	.225^**^	.135^**^	.180^**^	.210^**^	.181^**^	.133^**^	.154^**^	.204^**^	.305^**^	.303^**^	.135^**^	.238^**^	.384^**^	-	
17. Pros beha T3	1	4	2.84	0.52	.157^**^	.145^**^	.240^**^	.357^**^	.128^**^	.178^**^	.250^**^	.267^**^	.232^**^	.297^**^	.341^**^	.316^**^	.239^**^	.328^**^	.349^**^	.481^**^	-

*MR* meaningful roles, *CM* democratic classroom meetings, *Co* compliments, *aut* autonomy, *com* competence, *rel* relatedness, *Pros mot* Prosocial motivation, *Pros beha* Prosocial behavior. ^*^ *p* < .01, ^**^ *p* < .001

### Path Analyses

**Fig. 2:**
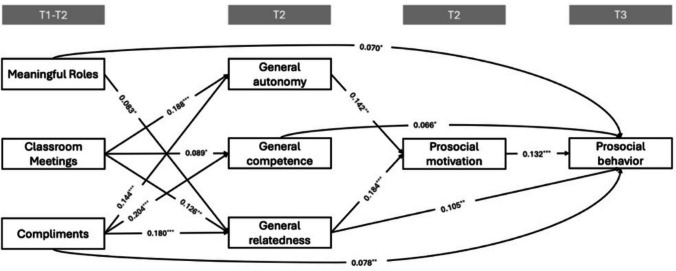
Path diagram of model 1: intervention components presence as independent variables**.** *Note.* Independent variables were assessed at T2 but refer to students’ experiences during the first half of the intervention (i.e., from T1 to T2). Non-significant associations, controlled variable (baseline prosocial behavior), and covariances at the same time point are not presented in the figure.^*^ *p* < .05, ^**^ *p* < .01, ^***^ *p* < .001

**Fig. 3:**
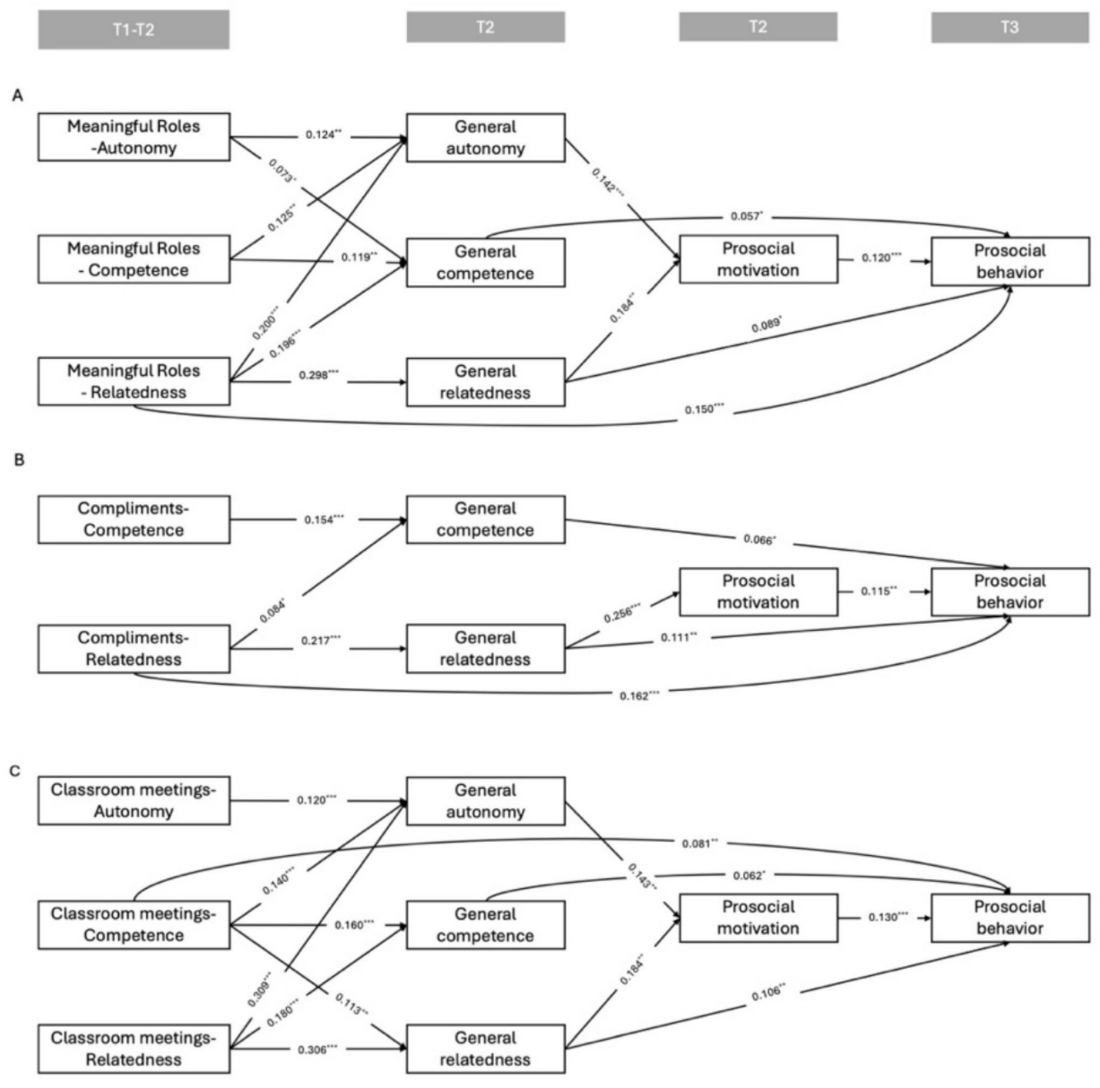
Path diagrams of model 2a-c: component-specific BPN fulfilment of meaningful roles (**A**), Compliments (**B**), and Classroom Meetings (**C**) as Independent Variables**.** *Note.* Independent variables were assessed at T2 but refer to students’ experiences during the first half of the intervention (i.e., from T1 to T2). Non-significant associations, controlled variable (baseline prosocial behavior), and covariances at the same time point are not presented in the figure. ^*^ *p* < .05, ^**^ *p* < .01, ^***^ *p* < .001

The final models demonstrated good fits to the data. See Table 2 for the model fit indices for each model. Table 3 summarizes the serial sequential mediation effects of each independent variable on prosocial behavior through BPN and prosocial motivation. Across models, general autonomy and general relatedness exhibited significant direct effects on prosocial motivation, whereas general competence did not; general competence and general relatedness showed significant longitudinal direct effects on prosocial behavior, whereas general autonomy did not. Prosocial motivation also showed significant longitudinal direct effects on prosocial behavior across all models. See Figs. 2 and 3 for all significant direct associations in the final models.

**Table 2:** Fit indices of the final models

Model	χ ^2^_SB_	*df*	CFI	RMSEA [90%CI]	SRMR
Model 1	0.784	3	1.000	0.000 [0.000, 0.028]	0.004
Model 2a	12.433	3	0.995	0.054 [0.025, 0.086]	0.016
Model 2b	37.704	2	0.976	0.128 [0.094, 0.165]^a^	0.039
Model 2c	4.076	3	0.999	0.018 [0.000, 0.057]	0.009

^a^RMSEA tends to be inflated when the degrees of freedom are low and when sample sizes are large, making the index overly sensitive to minor misfit. Considering that other indices of Model 2b all consistently indicate good model fit, we consider this acceptable

**Table 3:** Effects of independent variables on prosocial behavior via BPNs and prosocial behavior

Model	Path	Effect		Prosocial motivation Prosocial behavior				
		X	M1	β	SE	*p*	95% CI	
							Lower	Upper
Model 1	P1	MR	General autonomy	0.000	0.001	.480	−0.001	0.002
	P2		General competence	0.000	0.000	.956	0.000	0.000
	P3		General relatedness	0.002	0.001	.068	−0.001	0.005
	P4	Co	General autonomy	**0.003**	**0.001**	**.025**	**0.000**	**0.006**
	P5		General competence	0.000	0.001	.956	−0.002	0.002
	P6		General relatedness	**0.000**	**0.002**	**.029**	**0.000**	**0.008**
	P7	CM	General autonomy	**0.004**	**0.002**	**.036**	**0.000**	**0.007**
	P8		General competence	0.000	0.001	.956	−0.001	0.001
	P9		General relatedness	**0.003**	**0.002**	**.049**	**0.000**	**0.006**
Model 2a	P10	MR-autonomy	General autonomy	**0.002**	**0.001**	**.035**	**0.000**	**0.004**
	P11	MR-competence	General competence	0.000	0.001	.956	−0.001	0.001
	P12	MR-relatedness	General relatedness	**0.007**	**0.003**	**.011**	**0.001**	**0.012**
Model 2b	P13	Co-competence	General competence	0.000	0.001	.892	−0.001	0.002
	P14	Co-relatedness	General relatedness	**0.006**	**0.002**	**.005**	**0.002**	**0.011**
Model 2c	P15	CM-autonomy	General autonomy	0.002	0.001	.057	0.000	0.005
	P16	CM-competence	General competence	0.000	0.001	.956	−0.002	0.002
	P17	CM-relatedness	General relatedness	**0.007**	**0.003**	**.010**	**0.002**	**0.013**

*MR* Meaningful roles, *Co* Compliments, *CM* Democratic Classroom Meeting. Significant indirect paths are marked in bold

#### Intervention Components Quantity

The total effect of meaningful roles’ presence on prosocial behavior was significant (β = .084, *p* = .007) and operated primarily through a direct association; none of the indirect pathways reached significance. In contrast, democratic classroom meetings’ presence showed no significant total effect (β = .055, *p* = .082), yet specific indirect pathways through general relatedness and general autonomy were significant. For compliments’ presence, the total effect was also significant (β = .126, *p* < .001), and the indirect pathways through general relatedness and general autonomy were significant, while the direct effect remained significant (see Table 3 and Fig. 2).

#### Intervention Component Quality

Total effects showed that higher levels of component-specific relatedness fulfilment always predicted greater prosocial behavior (β = .107 to .198, *p* ≤ .001). Higher levels of compliment- and democratic classroom meeting-specific competence fulfilment also predicted greater prosocial behavior overall (β = .083 to .110, *p* ≤ .008), but not for meaningful roles. However, component-specific autonomy fulfilment showed no significant total effects. Moreover, component-specific BPN fulfilment was significantly associated with general BPN fulfilment for each component (Fig. 3A for meaningful roles, Fig. 3B for compliments, and Fig. 3C for democratic classroom meetings). Significant indirect effects were found for component-specific autonomy and relatedness fulfilment on prosocial behavior, mediated through their corresponding general BPNs and subsequent prosocial motivation. Specifically, the only autonomy-related indirect effect was found for role-specific autonomy, which showed a significant sequential pathway through general autonomy and prosocial motivation. For relatedness, all three component-specific relatedness indicators showed significant indirect effects via the sequential pathway involving general relatedness and prosocial motivation.

#### Sensitivity Analyses

To assess the robustness of the findings, we conducted sensitivity analyses by replacing the independent and mediating variables with those measured at T3. The overall pattern of results remained consistent. Particularly with respect to the sequential indirect pathways central to our hypotheses, the significance of all 17 tested indirect pathways remained the same. Detailed results are available in Supplementary Information 3. These findings suggest that the working mechanisms in the second half of the intervention were generally similar to those observed in the first half.

## Discussion

Prosocial behavior is essential for both individual well-being and societal cohesion. This study not only supported the idea that classroom-based strategies rooted in BPNT (Ryan & Deci, 2000b) can foster children’s prosocial behavior over time but also revealed *how* this occurs. Specifically, we found that prosocial behavior was enhanced through strategies that fulfil basic psychological needs and enhance prosocial motivation: Providing meaningful roles, encouraging peer compliments, and facilitating democratic classroom meetings, which are part of the Dutch Meaningful Roles intervention. Not only the presence of each intervention component (quantity), but also the degree to which each component fulfilled students’ basic psychological needs in its specific context (quality), was associated with their overall sense of need fulfilment and prosocial motivation, and, in turn, with higher prosocial behavior. These patterns were similar across both halves of the intervention period, indicating that these classroom-based strategies showed stable associations over time.

### Component-Specific Pathways to Prosocial Behavior

While studies have demonstrated the general effectiveness and the mechanisms of BPN-targeted intervention (e.g., Cheon et al., 2019; Ntoumanis et al., 2021), few studies have examined how specific components operate. Supporting the quantity pathways (H1), our findings disentangled the presence of three components, showing that although all promoted positive outcomes, they did so through distinct mechanisms.

Of all the components, the quantity of receiving compliments emerged as the most robust contributor to the fulfilment of all three psychological needs and prosocial motivation, and in turn, to prosocial behavior. Prior research showed that social rewards and emotional support particularly boosted social connection and reduced aggression (Ntoumanis et al., 2021), especially during late childhood and early adolescence when sensitivity to peer feedback intensifies (Lease et al., 2020). Moreover, receiving compliments boosted their sense of competence by encouraging a growth mindset—the belief that effort leads to improvement (Platte et al., 2025). Compliments presence supported autonomy as well; indeed, social rewards were previously shown to satisfy relatedness and autonomy needs (Ntoumanis et al., 2021). By fostering acceptance and belongingness (Vieno et al., 2007), compliments may have enabled students to internalize the prosocial value and act with their own volition and willingness to contribute to the group they belong to (Collie, 2022). Finally, compliments presence directly enhanced prosocial behavior beyond the mediation of basic psychological needs. Perhaps, receiving compliments generated positive affect and thereby also reinforced prosocial actions (Aknin et al., 2012).

Second, providing students with meaningful roles seemed a valuable strategy to support need fulfillment and prosocial behavior. Roles specifically contributed to general need fulfillment and prosocial motivation when students experienced autonomy or relatedness when carrying out their roles; simply assigning students a role was insufficient. Moreover, the effect of meaningful roles’ presence was not mediated by need fulfilment, suggesting alternative pathways. Perhaps, roles contribute to prosocial behavior through social skill acquisition (e.g., problem-solving, communication) rather than motivational pathways alone.

Last, the presence of democratic classroom meetings was also found to contribute to prosocial behavior, but only when they effectively fulfilled students’ needs for autonomy and relatedness. Their effects were fully mediated by general BPN fulfilment, consistent with findings that community-oriented practices, such as civic engagement activities, foster a sense of responsibility, peer connection, and competence (Gfroerer et al., 2024; Pavidis, 2023). When students feel accepted and safe, they are more likely to act according to their prosocial values (Ryan & Deci, 2000a, 2000b). Therefore, the effectiveness of democratic classroom meetings lies not in their presence alone, but in their capacity to fulfill core psychological needs.

Altogether, fulfilment of basic psychological needs seems not just a pathway to prosociality: It may be a prerequisite for meaningful and lasting behavioral change. Our results also highlight the value of examining individual intervention components rather than treating interventions as uniform wholes. Disentangling component-specific effects can inform the design of more targeted, adaptive, and effective programs.

### The Primacy of Relatedness in Fostering Prosocial Behavior

The results also supported the quality pathways (H2) and empirically demonstrate that enhancing BPN fulfillment within specific strategies can generalize to broader psychological need fulfillment and prosocial motivation, ultimately contributing to prosocial behavior. Out of the three basic psychological needs, relatedness appeared to be a particularly strong and consistent predictor of prosocial behavior. Experimental research similarly showed that participants’ prosociality was only enhanced when their sense of relatedness was manipulated, but not competence or autonomy (Pavey et al., 2011). This is consistent with BPNT’s proposition that satisfying a specific need may increase its perceived value and reinforce further engagement with experiences that fulfill that need (Moller et al., 2010; Ryan & Deci, 2000a, 2000b). Children may thus behave prosocially to deepen their sense of connection with others. This effect may be partly rooted in reciprocity: Children are more likely to help those they have relationships with or who have helped them before (Leimgruber, 2018). This suggests that relatedness may enhance prosociality, especially in reciprocal contexts. Interestingly, component-specific relatedness fulfilment also produced spillover effects on general autonomy and competence, supporting the BPNT claim that psychological needs are interrelated and mutually reinforcing (Vansteenkiste et al., 2020).

### Divergent Motivational Routes of Behavioral Change

Further, our results demonstrated evidence for the vital role of prosocial motivation when addressing BPN fulfillment to target behavioral change. However, this was exclusively the case for autonomy and relatedness, but not competence. Specifically, enhanced feelings of autonomy and relatedness were associated with prosocial behavior through increased motivation for such behavior. This aligns with the core hypothesis of the BPNT. However, students’ perceptions of their own competence predicted enhanced prosocial behavior directly through other, untested processes than motivation. Theoretically, this interpretation is plausible. Some skills gained during the intervention (e.g., expressing opinions in democratic classroom meetings or executing assigned tasks in meaningful roles) may benefit peers without reflecting a deliberate intention to act prosocially; rather, they may occur simply because children become more capable of helping others. In other words, students may, thus, feel more competent and use these skills to benefit others regardless of their own motivation to do so. Another, methodological, explanation may be that our measure reflected self-esteem more generally rather than *social* competence. Such domain-specific competence may translate more to students’ prosocial motivation.

### Limitations and Strengths

First, intervention components, general BPN fulfilment, and prosocial motivation were measured at T2, possibly inflating associations due to common method bias (Lindell & Whitney, 2001). However, intervention components were assessed retrospectively (the past few months), while the BPN fulfilment and motivation reflected current experiences. The sensitivity analyses using T3 data replicated the patterns without considerably larger effects (Supplementary Information 3), supporting the robustness of the findings. Nevertheless, it is difficult to determine causality. The longitudinal mediation model provides evidence consistent with our theoretical framework, yet alternative explanations such as maturation or bidirectional associations cannot be ruled out. Future research should establish causal effects by using controlled or experimental designs.

Second, self-reports, especially from children, may suffer from inaccuracies, including inconsistent recognition of components like democratic classroom meetings. Still, children’s perceived experiences are essential for evaluating implementation fidelity (Domitrovich et al., 2010). Furthermore, the prosocial behavior measure had a somewhat low reliability (α = 0.69). However, the items were significantly correlated with each other (*r* = .24–45).

Third, compliments were initially conceptualized as primarily supporting relatedness, and no autonomy-specific item was included. However, our findings suggest that receiving compliments may also foster autonomy-related experiences. Future work could address this by including autonomy-focused phrasing (e.g., “Receiving compliments makes me feel more comfortable/freer to act in my own way”) to better capture the impact of compliments.

Lastly, the two-wave, three-month design limits long-term inferences. That said, BPNT suggests motivation-based strategies have lasting developmental effects (Ryan & Deci, 2000a, 2000b), warranting further longitudinal research. Moreover, our assessment of intervention components did not include specific measures about the adherence of components, for instance quality of the compliments or adherence to democratic principles in the classroom meetings. Rather, it was measured somewhat indirectly by the BPN fulfilment. In future research, we will investigate the implementation of the program and analyze the qualitative data to get more insight into the quality of the program.

### Implications

This study highlights the importance of integrating strategies that fulfill students’ basic psychological needs into everyday classroom practice. Educators can flexibly apply individual components, such as assigning meaningful roles or encouraging compliments, to encourage prosociality. Beyond the studied strategies, teachers may also adopt other need-supportive approaches (e.g., autonomy-supportive teaching; Cheon et al., 2019) to create a prosocial classroom climate and facilitate students’ overall well-being.

Future research should examine not just if interventions work, but *how*—by testing changes in mediators and their impact on outcomes. While past intervention studies often emphasized skill-building strategies (e.g., Caprara et al., 2014), our findings highlight the promise of motivation-based interventions. Future studies should explore how to sustain prosociality by strengthening students’ intrinsic motivation through environments that fulfill their basic psychological needs. Further, it would be interesting to understand if some estimated associations between component experiences and prosocial motivation/behavior are transactional in nature. Especially for compliments, acting prosocial may not only result from receiving compliments but may also, vice versa, elicit more compliments – perhaps especially about behaviors that make them feel more related to others. Future research could examine those transactional associations between exposure to intervention components and BPN-related outcomes.

## Conclusion

This study shows that prosocial behavior in primary school-aged children may be fostered by fulfilling their basic psychological needs through classroom strategies. This study indicates that prosocial behavior in primary school-aged children may develop when their basic psychological needs are supported through classroom strategies. Carrying out meaningful roles, receiving growth-oriented compliments, and contributing to structured democratic classroom meetings were each associated with increases in children’s feelings of autonomy, competence, and relatedness over time, both within those specific activities and more generally. In turn, children reported feeling more motivated to act prosocially and also displayed more prosocial behavior. Intervention studies must go beyond studying main effects to identify *mechanisms* explaining how strategies can support children’s social-emotional development.

## Supplementary Information

Below is the link to the electronic supplementary material.

ESM 1 (DOCX 277 KB)

## Data Availability

Anonymized data, analysis code, and research materials are available by request to the corresponding author.

## Declarations

### Clinical Trial

This study is registered on ClinicalTrials.gov (Identifier: NCT05891067). The trial began on May 11, 2023, and was completed on July 31, 2024.

## References

[CR1] Aknin, L. B., Dunn, E. W., & Norton, M. I. (2012). Happiness runs in a circular motion: Evidence for a positive feedback loop between prosocial spending and happiness. *Journal of Happiness Studies,**13*(2), 347–355. 10.1007/s10902-011-9267-5

[CR2] Allen, M. S., Iliescu, D., & Greiff, S. (2022). Single item measures in psychological science: A call to action. *European Journal of Psychological Assessment,**38*(1), 1–5. 10.1027/1015-5759/a000699

[CR3] Angell, A. V. (2004). Making peace in elementary classrooms: A case for class meetings. *Theory & Research in Social Education,**32*(1), 98–104. 10.1080/00933104.2004.10473245

[CR4] Battistich, V., Solomon, D., Watson, M., & Schaps, E. (1997). Caring school communities. *Educational Psychologist,**32*(3), 137–151. 10.1207/s15326985ep3203_1

[CR5] Bollen, K. A. (1989). *Structural equations with latent variables* (pp. xiv, 514). John Wiley & Sons. 10.1002/9781118619179

[CR6] Boor-klip, H. J., Segers, E., Hendrickx, M. M. H. G., & Cillessen, A. H. N. (2014). Beleving van de peer context in de klas: Samenhang met sociaal functioneren, academisch functioneren en zelfbeeld. [Perceptions of classroom peer context: Associations with social status, academic achievement, and self-esteem.]. *Pedagogische Studiën,**91*(5), 288–301. https://pedagogischestudien.nl/article/view/14207

[CR7] Boor-klip, H. J., Segers, E., Hendrickx, M. M. H. G., & Cillessen, A. H. N. (2017). The moderating role of classroom descriptive norms in the association of student behavior with social preference and popularity. *Journal of Early Adolescence,**37*(3), 387–413. 10.1177/0272431615609158

[CR8] Caprara, G. V., Kanacri, B. P. L., Gerbino, M., Zuffianò, A., Alessandri, G., Vecchio, G., Caprara, E., Pastorelli, C., & Bridglall, B. (2014). Positive effects of promoting prosocial behavior in early adolescence: Evidence from a school-based intervention. *International Journal of Behavioral Development,**38*(4), 386–396. 10.1177/0165025414531464

[CR9] Caprara, G. V., Luengo Kanacri, B. P., Zuffianò, A., Gerbino, M., & Pastorelli, C. (2015). Why and how to promote adolescents’ prosocial behaviors: Direct, mediated and moderated effects of the cepidea school-based program. *Journal of Youth and Adolescence,**44*(12), 2211–2229. 10.1007/s10964-015-0293-1 25963445 10.1007/s10964-015-0293-1

[CR10] Caprara, G. V., & Steca, P. (2005). Self–efficacy beliefs as determinants of prosocial behavior conducive to life satisfaction across ages. *Journal of Social and Clinical Psychology,**24*(2), 191–217. 10.1521/jscp.24.2.191.62271

[CR11] Cheon, S. H., Reeve, J., & Ntoumanis, N. (2019). An intervention to help teachers establish a prosocial peer climate in physical education. *Learning and Instruction,**64*, Article 101223. 10.1016/j.learninstruc.2019.101223

[CR12] Collie, R. J. (2022). Social-emotional need satisfaction, prosocial motivation, and students’ positive behavioral and well-being outcomes. *Social Psychology of Education,**25*(2–3), 399–424. 10.1007/s11218-022-09691-w 35462752 10.1007/s11218-022-09691-w PMC9016699

[CR13] Collins, T. A., Drevon, D. D., Brown, A. M., Villarreal, J. N., Newman, C. L., & Endres, B. (2020). Say something nice: A meta-analytic review of peer reporting interventions. *Journal of School Psychology,**83*, 89–103. 10.1016/j.jsp.2020.10.002 33276857 10.1016/j.jsp.2020.10.002

[CR14] Cong, C. W., & Cheong, J. Y. (2023). Validation of Rosenberg self-esteem scale for Malaysian adolescents. *Current Psychology,**42*(21), 17835–17838. 10.1007/s12144-022-02960-z

[CR15] Domitrovich, C. E., Bradshaw, C. P., Greenberg, M. T., Embry, D., Poduska, J. M., & Ialongo, N. S. (2010). Integrated models of school-based prevention: Logic and theory. *Psychology in the Schools,**47*(1), 71–88. 10.1002/pits.20452 27182089 10.1002/pits.20452 PMC4865396

[CR16] Eisenberg, N., Spinrad, T. L., & Knafo-Noam, A. (2015). Prosocial development. In *Handbook of Child Psychology and Developmental Science* (pp. 1–47). John Wiley & Sons, Ltd. 10.1002/9781118963418.childpsy315

[CR17] Ellis, B. J., Del Giudice, M., Dishion, T. J., Figueredo, A. J., Gray, P., Griskevicius, V., Hawley, P. H., Jacobs, W. J., James, J., Volk, A. A., & Wilson, D. S. (2012). The evolutionary basis of risky adolescent behavior: Implications for science, policy, and practice. *Developmental Psychology,**48*(3), 598–623. 10.1037/a0026220 22122473 10.1037/a0026220

[CR18] Ellis, B. J., Volk, A. A., Gonzalez, J.-M., & Embry, D. D. (2016). The meaningful roles intervention: An evolutionary approach to reducing bullying and increasing prosocial behavior. *Journal of Research on Adolescence,**26*(4), 622–637. 10.1111/jora.12243 28453200 10.1111/jora.12243

[CR19] Franck, E., Raedt, R. D., Barbez, C., & Rosseel, Y. (2008). Psychometric properties of the Dutch Rosenberg Self-Esteem Scale. *Psychologica Belgica*. 10.5334/pb-48-1-25

[CR20] Freitas, I. D. S., Oliveira, G. E. D., Lima, V. D. S., & Melo, M. H. D. S. (2021). Evidence-based interventions for promoting prosocial behavior in schools: Integrative review. *Psicologia - Teoria e Prática*, *23*(3). 10.5935/1980-6906/ePTPPE14091

[CR21] Gfroerer, K., Edwards, D., & Dwight, E. (2024). Utilizing class meetings to teach essential social-emotional skills and build community. *The Journal of Individual Psychology,**80*(3), 188–202.

[CR22] Hu, L., & Bentler, P. M. (1999). Cutoff criteria for fit indexes in covariance structure analysis: Conventional criteria versus new alternatives. *Structural Equation Modeling: A Multidisciplinary Journal,**6*(1), 1–55. 10.1080/10705519909540118

[CR23] Jackson, C. K., Porter, S. C., Easton, J. Q., Blanchard, A., & Kiguel, S. (2020). School effects on socioemotional development, school-based arrests, and educational attainment. *American Economic Review: Insights,**2*(4), 491–508. 10.1257/aeri.20200029

[CR24] Lease, A. M., Kwon, K., Lovelace, M., & Huang, H. (2020). Peer influence in elementary school: The importance of assessing the likeability of popular children. *The Journal of Genetic Psychology,**181*(2–3), 95–110. 10.1080/00221325.2020.1730744 32090707 10.1080/00221325.2020.1730744

[CR25] Leimgruber, K. L. (2018). The developmental emergence of direct reciprocity and its influence on prosocial behavior. *Current Opinion in Psychology,**20*, 122–126. 10.1016/j.copsyc.2018.01.006 29486397 10.1016/j.copsyc.2018.01.006

[CR26] Lindell, M. K., & Whitney, D. J. (2001). Accounting for common method variance in cross-sectional research designs. *Journal of Applied Psychology,**86*(1), 114–121. 10.1037/0021-9010.86.1.114 11302223 10.1037/0021-9010.86.1.114

[CR27] Manzano-Sánchez, D., Gómez-Mármol, A., Valero-Valenzuela, A., & Jiménez-Parra, J. F. (2021). School climate and responsibility as predictors of antisocial and prosocial behaviors and violence: A study towards self-determination theory. *Behavioral Sciences,**11*(3), Article 36. 10.3390/bs11030036 33802667 10.3390/bs11030036 PMC8002525

[CR28] Matthews, R. A., Pineault, L., & Hong, Y.-H. (2022). Normalizing the use of single-item measures: Validation of the single-item compendium for organizational psychology. *Journal of Business and Psychology,**37*(4), 639–673. 10.1007/s10869-022-09813-3

[CR29] Maydeu-Olivares, A. (2017). Maximum likelihood estimation of structural equation models for continuous data: Standard errors and goodness of fit. *Structural Equation Modeling: A Multidisciplinary Journal,**24*(3), 383–394. 10.1080/10705511.2016.1269606

[CR30] Mesurado, B., Guerra, P., Richaud, M. C., & Rodriguez, L. M. (2019). Effectiveness of prosocial behavior interventions: A meta-analysis. *Psychiatry and Neuroscience Update: From Translational Research to a Humanistic Approach—Volume III* (pp. 259–271). Springer International Publishing. 10.1007/978-3-319-95360-1_21

[CR31] Moller, A. C., Deci, E. L., & Elliot, A. J. (2010). Person-level relatedness and the incremental value of relating. *Personality and Social Psychology Bulletin,**36*(6), 754–767. 10.1177/0146167210371622 20460651 10.1177/0146167210371622

[CR32] Muthén, L.K. and Muthén, B.O. (1998–2017). *Mplus User’s Guide. Eighth Edition.* Los Angeles, CA: Muthén & Muthén

[CR33] Ntoumanis, N., Ng, J. Y. Y., Prestwich, A., Quested, E., Hancox, J. E., Thøgersen-Ntoumani, C., Deci, E. L., Ryan, R. M., Lonsdale, C., & Williams, G. C. (2021). A meta-analysis of self-determination theory-informed intervention studies in the health domain: Effects on motivation, health behavior, physical, and psychological health. *Health Psychology Review,**15*(2), 214–244. 10.1080/17437199.2020.1718529 31983293 10.1080/17437199.2020.1718529

[CR34] Okun, M. A., Yeung, E. W., & Brown, S. (2013). Volunteering by older adults and risk of mortality: A meta-analysis. *Psychology and Aging,**28*(2), 564–577. 10.1037/a0031519 23421326 10.1037/a0031519

[CR35] Pauw, L. M. J. (2013). *Onderwijs en burgerschap: Wat vermag de basisschool? Onderzoek naar De Vreedzame School* [Dissertation]. Utrecht University. Retrieved January 26, 2025, from https://dspace.library.uu.nl/handle/1874/276048

[CR36] Pavey, L., Greitemeyer, T., & Sparks, P. (2011). Highlighting relatedness promotes prosocial motives and behavior. *Personality and Social Psychology Bulletin,**37*(7), 905–917. 10.1177/0146167211405994 21521720 10.1177/0146167211405994

[CR37] Pavidis, K. A. (2023). *Mixed-methods study of the impact of regularly implemented class meetings on elementary school students’ academic engagement, behavior, and class & school culture* (Order No. 30813972). [Doctoral dissertation, Concordia University Irvine]. ProQuest. Retrived March 1, 2025, from https://www.proquest.com/dissertations-theses/mixed-methods-study-impact-regularly-implemented/docview/2898270472/se-2

[CR38] Platte, D., Xu, K. M., & de Groot, R. H. M. (2025). The effect of fostering a growth mindset in primary school children: Does intervention approach matter? *Education Sciences,**15*(3), Article 3. 10.3390/educsci15030327

[CR39] Poulin, M. J., & Holman, E. A. (2013). Helping hands, healthy body? Oxytocin receptor gene and prosocial behavior interact to buffer the association between stress and physical health. *Hormones and Behavior,**63*(3), 510–517. 10.1016/j.yhbeh.2013.01.004 23354128 10.1016/j.yhbeh.2013.01.004

[CR40] Reeve, J., & Jang, H. (2006). What teachers say and do to support students’ autonomy during a learning activity. *Journal of Educational Psychology,**98*(1), 209–218. 10.1037/0022-0663.98.1.209

[CR41] Ryan, R. M. (2023). *The Oxford Handbook of Self-determination Theory*. Oxford University Press. 10.1093/oxfordhb/9780197600047.001.0001

[CR42] Ryan, R. M., & Deci, E. L. (2000a). Intrinsic and extrinsic motivations: Classic definitions and new directions. *Contemporary Educational Psychology*. 10.1006/ceps.1999.1020 10620381 10.1006/ceps.1999.1020

[CR43] Ryan, R. M., & Deci, E. L. (2000b). Self-determination theory and the facilitation of intrinsic motivation, social development, and well-being. *American Psychologist,**55*(1), 68–78. 10.1037/0003-066X.55.1.68 11392867 10.1037//0003-066x.55.1.68

[CR44] Sheldon, K. M., Ryan, R., & Reis, H. T. (1996). What makes for a good day? Competence and autonomy in the day and in the person. *Personality and Social Psychology Bulletin,**22*(12), 1270–1279. 10.1177/01461672962212007

[CR45] van Loon, A. W. G., & Kaufman, T. M. L. (2023). The effectiveness of the Dutch Meaningful Roles program in children: A study protocol for a cluster randomized controlled study. *BMC Public Health,**23*(1), Article 1440. 10.1186/s12889-023-16362-8 37501078 10.1186/s12889-023-16362-8 PMC10375606

[CR46] van Widenfelt, B. M., Goedhart, A. W., Treffers, P. D. A., & Goodman, R. (2003). Dutch version of the Strengths and Difficulties Questionnaire (SDQ). *European Child & Adolescent Psychiatry,**12*(6), 281–289. 10.1007/s00787-003-0341-3 14689260 10.1007/s00787-003-0341-3

[CR47] Vansteenkiste, M., Ryan, R. M., & Soenens, B. (2020). Basic psychological need theory: Advancements, critical themes, and future directions. *Motivation and Emotion,**44*(1), 1–31. 10.1007/s11031-019-09818-1

[CR48] Vaz, S., Falkmer, M., Ciccarelli, M., Passmore, A., Parsons, R., Tan, T., & Falkmer, T. (2015). The personal and contextual contributors to school belongingness among primary school students. *PLoS ONE,**10*(4), Article e0123353. 10.1371/journal.pone.0123353 25876074 10.1371/journal.pone.0123353 PMC4398482

[CR49] Vieno, A., Santinello, M., Pastore, M., & Perkins, D. D. (2007). Social support, sense of community in school, and self-efficacy as resources during early adolescence: An integrative model. *American Journal of Community Psychology,**39*(1), 177–190. 10.1007/s10464-007-9095-2 17437191 10.1007/s10464-007-9095-2

[CR50] Volk, A. A., Dane, A. V., & Marini, Z. A. (2014). What is bullying? A theoretical redefinition. *Developmental Review,**34*(4), 327–343. 10.1016/j.dr.2014.09.001

[CR51] Wray-Lake, L., & Syvertsen, A. K. (2011). The developmental roots of social responsibility in childhood and adolescence. *New Directions for Child and Adolescent Development,**2011*(134), 11–25. 10.1002/cd.308 22147598 10.1002/cd.308

