# Supplementary material for: Pathways to Prosociality: How Classroom Strategies That Support Basic Psychological Needs Foster Prosocial Behavior in Children
Source: Prev Sci. 2026 Mar 18;27(3):451–63. doi: 10.1007/s11121-026-01895-2 (PMC13102884; doi:10.1007/s11121-026-01895-2)
Supplement: Supplementary file 1 — (DOCX 277 KB) [file 11121_2026_1895_MOESM1_ESM.docx]

**Supplementary Information**

**Article title**: Pathways to Prosociality: How Classroom Strategies That Support Basic Psychological Needs Foster Prosocial Behavior in Children

**Journal name**: Prevention Science

**Author names**: Yue Sun, Amanda W. G. van Loon & Tessa M. L. Kaufman

**Affiliation and e-mail address of the corresponding author**: Yue Sun;

University of Amsterdam. Research Institute for Child Development and Education. Nieuwe Achtergracht 127, 1018WS Amsterdam, the Netherlands; [y.sun2@uva.nl](mailto:y.sun2@uva.nl).

**Supplementary Information 1**

**Table S1.**

*Descriptive Statistics for Study Variables at T2 per School*

|  | MR | MR aut | MR com | MR rel | CM | CM aut | CM com | CM rel | Co | Co com | Co rel | Gen aut | Gen com | Gen rel | Pros beh |
| --- | --- | --- | --- | --- | --- | --- | --- | --- | --- | --- | --- | --- | --- | --- | --- |
| School 1 (*N* = 66) | 2.55 (2.55) | 2.79 (1.03) | 3.43 (0.75) | 2.92 (0.81) | 2.80 (1.61) | 3.25 (0.92) | 2.89 (0.85) | 3.13 (0.63) | 2.80 (1.24) | 3.08 (0.96) | 2.90 (1.01) | 3.09 (0.42) | 2.66 (0.38) | 4.18 (0.61) | 2.91 (0.49) |
| School 2 (*N* = 37) | 1.72 (2.21) | 2.37 (1.17) | 3.16 (0.96) | 2.95 (1.08) | 1.06 (1.39) | 2.67 (0.97) | 2.44 (0.98) | 2.61 (0.78) | 3.17 (1.84) | 3.27 (0.87) | 3.30 (0.84) | 2.72 (0.55) | 2.54 (0.47) | 3.94 (0.79) | 2.66 (0.55) |
| School 3 (*N* = 52) | 2.92 (1.57) | 2.80 (1.04) | 3.13 (1.08) | 2.56 (1.18) | 3.63 (0.95) | 3.29 (0.94) | 2.71 (1.17) | 3.06 (0.93) | 3.18 (1.42) | 2.96 (1.07) | 2.83 (1.06) | 3.07 (0.59) | 2.52 (0.45) | 3.90 (0.98) | 2.65 (0.58) |
| School 4 (*N* = 138) | 2.11 (2.00) | 2.75 (0.96) | 3.13 (0.80) | 2.78 (0.99) | 2.82 (1.53) | 3.15 (0.98) | 2.76 (0.92) | 2.96 (0.72) | 2.76 (1.40) | 3.16 (0.89) | 2.95 (1.05) | 2.95 (0.51) | 2.63 (0.37) | 3.99 (0.67) | 2.84 (0.45) |
| School 5 (*N* = 108) | 2.22 (1.99) | 2.66 (1.04) | 3.18 (0.80) | 2.70 (1.03) | 1.48 (1.56) | 2.23 (1.11) | 2.72 (0.92) | 2.84 (0.82) | 2.88 (1.60) | 2.82 (0.91) | 2.77 (0.93) | 2.85 (0.50) | 2.56 (0.40) | 3.91 (0.74) | 2.79 (0.51) |
| School 6 (*N* = 46) | 3.28 (1.41) | 2.66 (1.06) | 3.34 (0.83) | 3.25 (0.94) | 3.07 (1.25) | 3.59 (0.73) | 3.05 (1.01) | 3.52 (0.70) | 3.04 (1.51) | 3.35 (0.78) | 3.02 (0.99) | 3.11 (0.51) | 2.75 (0.30) | 4.60 (0.39) | 2.89 (0.61) |
| School 7 (*N* = 36) | 2.94 (1.88) | 2.57 (0.92) | 2.96 (0.96) | 2.57 (1.17) | 1.61 (1.76) | 3.15 (1.14) | 2.40 (1.10) | 2.55 (0.83) | 1.94 (1.87) | 2.86 (1.11) | 2.57 (1.12) | 2.52 (0.59) | 2.54 (0.47) | 3.73 (0.89) | 2.63 (0.70) |
| School 8 (*N* = 57) | 1.35 (1.93) | 2.64 (1.09) | 2.95 (0.79) | 2.68 (0.89) | 1.67 (1.67) | 2.57 (1.26) | 2.27 (0.96) | 2.54 (0.65) | 3.00 (1.54) | 3.08 (0.80) | 2.61 (0.87) | 2.69 (0.43) | 2.49 (0.42) | 3.69 (0.69) | 2.77 (0.43) |
| School 9 (*N* = 122) | 3.06 (1.72) | 2.76 (1.01) | 3.30 (0.70) | 2.85 (1.00) | 3.66 (0.92) | 3.44 (0.78) | 2.92 (0.87) | 3.06 (0.72) | 2.59 (1.57) | 3.05 (0.95) | 2.91 (0.95) | 3.16 (0.50) | 2.63 (0.41) | 4.31 (0.66) | 3.08 (0.48) |
| School 10 (*N* = 74) | 3.26 (1.85) | 2.88 (1.08) | 3.36 (0.74) | 3.00 (0.95) | 1.75 (1.51) | 2.96 (1.02) | 2.76 (0.98) | 3.24 (0.68) | 2.88 (1.70) | 3.22 (0.91) | 2.97 (0.96) | 3.05 (0.49) | 2.73 (0.32) | 4.25 (0.63) | 2.95 (0.49) |
| School 11 (*N* = 65) | 2.05 (1.67) | 2.89 (0.98) | 3.19 (0.80) | 2.77 (0.94) | 3.20 (1.16) | 3.22 (0.87) | 3.00 (0.86) | 2.76 (0.80) | 2.15 (1.35) | 3.02 (0.90) | 2.96 (0.89) | 2.89 (0.48) | 2.44 (0.52) | 3.77 (0.81) | 2.82 (0.51) |
| School 12 (*N* = 79) | 3.03 (1.01) | 2.99 (0.85) | 3.37 (0.63) | 2.83 (1.04) | 3.63 (0.70) | 3.15 (0.86) | 2.91 (0.84) | 2.89 (0.64) | 1.61 (1.15) | 3.07 (0.92) | 2.77 (0.89) | 2.95 (0.47) | 2.58 (0.42) | 4.14 (0.72) | 2.79 (0.47) |
| School 13 (*N* = 13) | 1.17 (1.64) | 3.00 (0.71) | 2.80 (0.84) | 2.60 (0.89) | 1.75 (1.66) | 2.13 (1.25) | 3.38 (0.74) | 3.13 (0.64) | 3.67 (1.61) | 2.73 (1.10) | 2.27 (0.79) | 2.95 (0.55) | 2.75 (0.27) | 4.10 (0.56) | 2.81 (0.38) |
| School 14 (*N* = 13) | 3.33 (1.72) | 1.91 (0.94) | 3.00 (0.78) | 2.27 (1.19) | 2.42 (1.24) | 3.25 (0.97) | 2.17 (0.84) | 2.50 (0.80) | 3.00 (1.76) | 2.60 (1.35) | 2.70 (1.06) | 2.75 (0.48) | 2.37 (0.35) | 3.85 (0.53) | 2.35 (0.59) |
| School 15 (*N* = 44) | 3.02 (1.56) | 2.74 (1.04) | 3.14 (0.84) | 2.81 (1.11) | 2.75 (1.28) | 3.02 (1.04) | 2.78 (1.04) | 3.02 (0.88) | 2.84 (1.63) | 3.21 (0.94) | 2.95 (0.96) | 2.92 (0.56) | 2.55 (0.47) | 4.20 (0.73) | 2.95 (0.58) |
| School 16 (*N* = 40) | 2.08 (2.03) | 2.52 (0.92) | 2.80 (0.82) | 2.52 (0.96) | 2.35 (1.72) | 2.94 (0.85) | 2.71 (0.97) | 2.77 (0.67) | 2.03 (1.51) | 2.84 (1.04) | 2.77 (1.06) | 2.76 (0.47) | 2.52 (0.51) | 3.84 (0.87) | 2.63 (0.50) |
| School 17 (*N* = 58) | 2.35 (1.82) | 2.92 (0.92) | 3.38 (0.79) | 3.46 (0.73) | 3.07 (1.21) | 3.29 (0.76) | 3.04 (0.92) | 3.13 (0.64) | 3.02 (1.48) | 3.06 (0.93) | 3.00 (0.98) | 3.03 (0.56) | 2.64 (0.41) | 4.22 (0.78) | 2.87 (0.53) |
| School 18 (*N* = 52) | 3.04 (1.73) | 2.37 (0.92) | 2.95 (0.74) | 2.46 (0.90) | 2.98 (1.27) | 3.04 (1.01) | 2.65 (0.86) | 2.71 (0.80) | 1.80 (1.21) | 2.72 (0.85) | 2.37 (0.77) | 2.70 (0.55) | 2.41 (0.49) | 3.71 (0.76) | 2.76 (0.41) |
| School 19 (*N* = 22) | 4.14 (1.21) | 3.09 (0.75) | 3.36 (0.66) | 3.59 (0.50) | 3.95 (0.73) | 3.59 (0.73) | 2.91 (0.92) | 3.40 (0.82) | 2.95 (1.05) | 3.14 (0.38) | 2.68 (0.30) | 3.14 (0.38) | 2.68 (0.30) | 4.45 (0.54) | 3.15 (0.47) |

*Note.* MR = Meaningful Roles, CM = Democratic Classroom Meetings, Co = Compliments, aut = Autonomy, com = Competence, rel = Relatedness, Pros beha = Prosocial Behavior.

**Supplementary Information 2**

The analyses deviated from the preregistration in two ways. First, given the relatively modest explained variance at the classroom level for prosocial behavior (7.7%) and the poor model fit in classroom-level analyses (e.g., high RMSEA values ranging from 0.098 to 0.195 and low TLI values between 0.217 and 0.853), classroom-level structure did not substantially improve model estimation. Therefore, we conducted analyses at the individual level. This decision was further supported by the predominance of individual-level associations and the fact that all measures were collected at this level. Second, the intrinsic prosocial motivation is included in the models as a second mediator rather than a separate prosociality indicator to better present the theoretical framework.

**Supplementary Information 3**

To examine whether the working mechanisms in the second half of the intervention mirrored those in the first phase, we replaced the original independent and mediating variables with intervention components, general BPNs, and intrinsic prosocial motivation measured at T3. Compared with the original models, only minor changes were observed. In Model 1, the direct path from general autonomy to prosocial behavior became significant, while the path from general competence to prosocial behavior was no longer significant. In Model 2a, the direct effect of meaningful roles-specific autonomy fulfilment on prosocial behavior became significant, whereas the paths from meaningful roles-specific competence fulfilment to general autonomy and general relatedness became non-significant. No changes were found in the significance levels of any paths in Models 2b and 2c. See the model diagrams and the direct effects in Figures S3.1 and S3.2. The model fit indices were summarised in Table S3.1, and the estimations of indirect effects were summarised in Table S3.2

**Figure S3.1**

*Path Diagram of Model 1: Intervention Components Presence as Independent Variables, T3 Measurements*


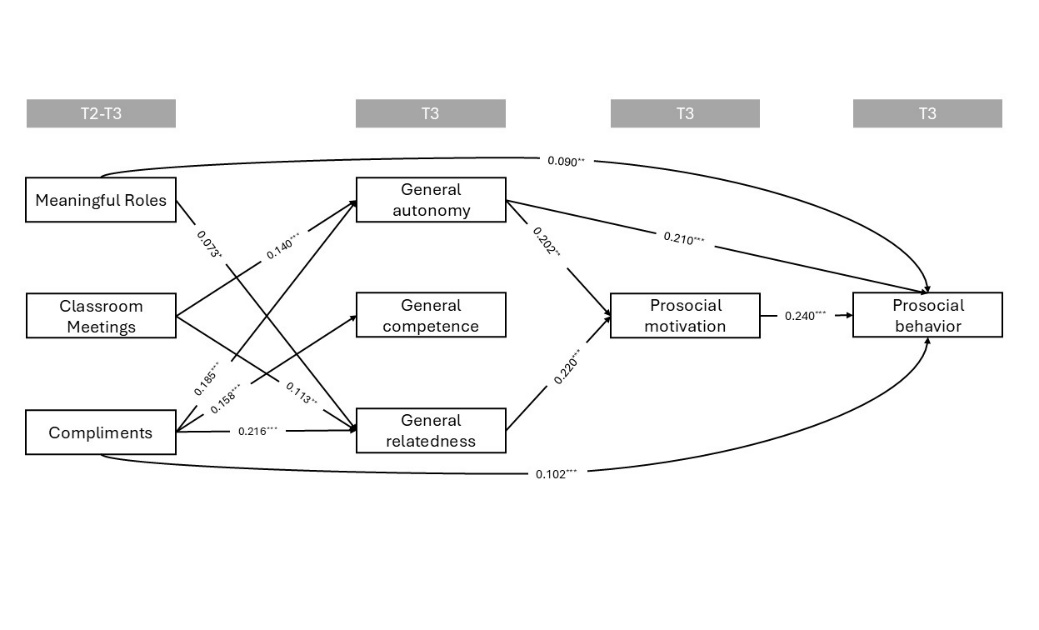


*Note.* Independent variables were assessed at T2 but refer to students’ experiences during the first half of the intervention (i.e., from T1 to T2). Non-significant associations, controlled variable (baseline prosocial behavior), and covariances at the same time point are not presented in the figure. ^*^ *p* < .05, ^**^ *p* < .01, *^***^* p < .001.

**Figure S3.2**

*Path Diagrams of Model 2a-c: Component-specific BPN Fulfilment of Meaningful Roles (A), Compliments (B), and Classroom Meetings (C) as Independent Variables, T3 Measurements*


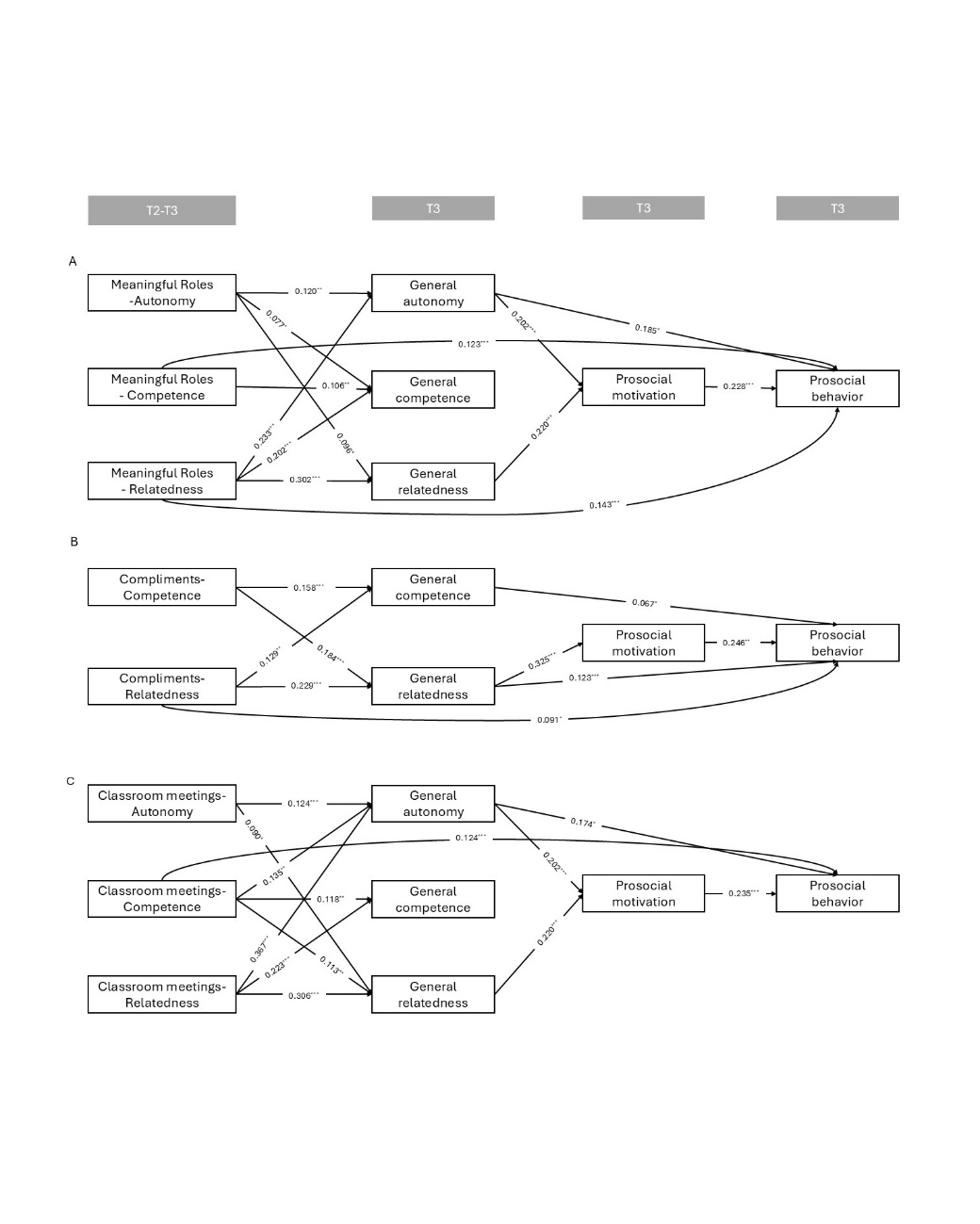


*Note.* Independent variables were assessed at T2 but refer to students’ experiences during the first half of the intervention (i.e., from T1 to T2). Non-significant associations, controlled variable (baseline prosocial behavior), and covariances at the same time point are not presented in the figure.^*^ *p* < .05, ^**^ *p* < .01, *^***^* p < .001.

**Table 3.1**

*Fit Indices of the Final Models*

| Model | χ ^2^ _SB_ | *df* | CFI | RMSEA [90%CI] | SRMR |
| --- | --- | --- | --- | --- | --- |
| Model 1 | 0.340 | 3 | 1.000 | 0.000 [0.000, 0.000] | 0.003 |
| Model 2a | 8.106 | 3 | 0.998 | 0.040 [0.006, 0.074] | 0.011 |
| Model 2b | 52.389 | 2 | 0.967 | 0.156 [0.122, 0.193]^a^ | 0.049 |
| Model 2c | 4.316 | 3 | 0.999 | 0.020 [0.000, 0.059] | 0.009 |

*Note.* All *N* = 1082. ^a^RMSEA tends to be inflated when the degrees of freedom are low and when sample sizes are large, making the index overly sensitive to minor misfit. Considering that other indices of Model 2b all consistently indicate good model fit, we consider this acceptable.

**Table 3.2**

*Standardized Effects of Independent Variables on Prosocial Behavior via BPNs and Prosocial Behavior at T3*

| Model | Path | Effect | | X 🡪 M1🡪 Prosocial motivation 🡪 Prosocial behavior | | | | |
| --- | --- | --- | --- | --- | --- | --- | --- | --- |
|  |  | X | M1 | β | SE | *p* | 95% CI | |
|  |  |  |  |  |  |  | Lower | Upper |
| Model 1 | P1 | MR | General autonomy | 0.003 | 0.002 | .099 | -0.002 | 0.006 |
|  | P2 |  | General competence | 0.000 | 0.001 | .475 | -0.000 | 0.001 |
|  | P3 |  | General relatedness | 0.004 | 0.002 | .083 | 0.000 | 0.008 |
|  | P4 | Co | General autonomy | **0.009** | **0.003** | **.001** | **0.004** | **0.014** |
|  | P5 |  | General competence | 0.000 | 0.001 | .956 | -0.004 | 0.002 |
|  | P6 |  | General relatedness | **0.011** | **0.003** | **< .001** | **0.006** | **0.017** |
|  | P7 | CM | General autonomy | **0.007** | **0.003** | **.010** | **0.002** | **0.012** |
|  | P8 |  | General competence | 0.000 | 0.000 | .839 | -0.001 | 0.000 |
|  | P9 |  | General relatedness | **0.006** | **0.002** | **.012** | **0.001** | **0.011** |
| Model 2a | P10 | MR-autonomy | General autonomy | **0.006** | **0.003** | **.038** | **0.000** | **0.006** |
|  | P11 | MR-competence | General competence | -0.000 | 0.001 | .455 | -0.003 | 0.003 |
|  | P12 | MR-relatedness | General relatedness | **0.015** | **0.004** | **< .001** | **0.004** | **0.012** |
| Model 2b | P13 | Co-competence | General competence | -0.000 | 0.001 | .734 | -0.001 | 0.002 |
|  | P14 | Co-relatedness | General relatedness | **0.018** | **0.005** | **.001** | **0.002** | **0.011** |
| Model 2c | P15 | CM-autonomy | General autonomy | **0.006** | **0.002** | **.008** | **0.001** | **0.006** |
|  | P16 | CM-competence | General competence | -0.001 | 0.001 | .447 | -0.002 | 0.001 |
|  | P17 | CM-relatedness | General relatedness | **0.017** | **0.005** | **< .001** | **0.006** | **0.018** |

*Note.* MR = Meaningful roles, Co = Compliments, CM = Democratic Classroom Meeting. Significant indirect paths are marked in bold.
